# Theoretical luminescence spectra in p-type superlattices based on InGaAsN

**DOI:** 10.1186/1556-276X-7-607

**Published:** 2012-10-31

**Authors:** Thiago F de Oliveira, Sara CP Rodrigues, Luísa MR Scolfaro, Guilherme M Sipahi, Eronides F da Silva

**Affiliations:** 1 Departamento de Física, Universidade Federal Rural de Pernambuco, Rua Dom Manoel de Medeiro s/n, Recife, Pernambuco, 52171-900, Brazil; 2 Department of Physics, Texas State University, San Marcos, TX, 78666, USA; 3 Instituto de Física de São Carlos, Universidade de São Paulo, CP369, São Carlos, São Paulo, 13560-970, Brazil; 4 Departamento de Física, Universidade Federal de Pernambuco, Cidade Universitária, Pernambuco, 50670-901, Brazil

**Keywords:** Dilute nitride semiconductor, Luminescence, $\vec{k}\;\vec{p}$ method, p-doped, Nanostructures

## Abstract

In this work, we present a theoretical photoluminescence (PL) for p-doped GaAs/InGaAsN nanostructures arrays. We apply a self-consistent $\vec{k}\;\vec{p}$ method in the framework of the effective mass theory. Solving a full 8 × 8 Kane's Hamiltonian, generalized to treat different materials in conjunction with the Poisson equation, we calculate the optical properties of these systems. The trends in the calculated PL spectra, due to many-body effects within the quasi-two-dimensional hole gas, are analyzed as a function of the acceptor doping concentration and the well width. Effects of temperature in the PL spectra are also investigated. This is the first attempt to show theoretical luminescence spectra for GaAs/InGaAsN nanostructures and can be used as a guide for the design of nanostructured devices such as optoelectronic devices, solar cells, and others.

## Background

In the last decade, the study of quaternary InGaAsN alloy systems has attracted a great deal of attention due to its potential application in nanostructured devices such as next-generation multijunction solar cells and optoelectronic devices for optical communications [1-5]. Incorporation of a small amount of nitrogen (<2%) to InGaAs reduces the net strain because of the smaller atomic size of nitrogen (0.75 Å) compared with arsenic (1.33 Å), decreasing the bandgap due to a large bandgap bowing [6]. Therefore, by carefully controlling the composition ratios, one should be able to achieve InGaAsN epitaxial layers lattice-matched to GaAs substrates [7]. The use of these alloys in the manufacture of laser regions for optical communication emitting at the range of 1.3 to 1.5 μm shows several advantages, e.g., it has been demonstrated to be a low-cost replacement for directly modulated 1.3-μm InP devices used in network applications as wireless access points and Ethernet switches [8,9]. In addition, the diluted quaternary nitride alloys are of great interest for high-conversion efficiency solar cells and heterojunction bipolar transistors (HBT) with low turn-on voltage for portable devices [2-5]. For space photovoltaic applications, high-efficiency solar cell are advantageous for increasing the available electrical power or alternately reducing satellite mass and launch cost [2].

In order to improve the development of new dilute nitride-based devices, it is important to investigate the photoluminescence (PL) properties of semiconductor nanostructures [10]. Although an investigation on the PL properties of p-type-doped InGaAsN systems is of particular interest due to its potential usage in n-p-n HBT devices as the base layer [11-15], few reports are found on the literature. Generally, beryllium has been used as the p-type dopant in the InGaAsN layers [10,11]. From an experimental point of view, rapid thermal annealing (RTA) has been demonstrated to improve the PL intensity and the internal quantum efficiency of solar cells [6]. The real importance of this technique is that RTA can effectively reduce the composition fluctuation and suppress the InGaAs-rich phase [16]. This fact was also observed in GaAsN alloys, confirming the formation of localized states inside the wells [17].

In this work, we investigate the theoretical PL spectra calculations for p-doped GaAs/InGaAsN nanostructures. The calculations are performed within the $\vec{k}\;\vec{p}$ method by solving the full 8 × 8 Kane's Hamiltonian, generalized to treat different materials. Strain effects due to the lattice mismatch between InGaAsN and GaAs are also taken into account. By varying the acceptor concentration and well width, we analyze the effect of exchange-correlation, which plays an important role in the potential profile and electronic transitions. We also investigate the effects of temperature in the PL spectra. These results can explain several important aspects on the optical properties of these nanostructured systems.

## Methods

The calculations are carried out by solving the 8 × 8 Kane's multiband effective mass equation (EME) which is represented with respect to a basis set of plane waves. We assume an infinite superlattice (SL) of squared well along the <001 > direction. The multiband EME is represented with respect to the plane waves with the wave vectors, *K* = (2*π*/*d*)*l* (*l* is an integer), equal to the reciprocal SL vectors. Rows and columns of the 8 × 8 Kane's Hamiltonian refer to the Bloch-type eigenfunctions $|jm_{j}\vec{k}\rangle$ of the Γ_8_ heavy and light hole bands, Γ_7_ spin-orbit hole bands, and Γ_6_ electron bands; $\vec{k}$ denotes a vector of the first Brillouin zone.

Expanding the EME with respect to the plane waves 〈*z*|*K*〉 means representing this equation in terms of the Bloch function $\langle \vec{r}|jm_{j}\vec{k}+K{\vec{e}}_{z}\rangle$. For a Bloch function $\langle z|E\vec{k}\rangle$ of the SL corresponding to energy *E* and the wave vector $\vec{k}$, the EME takes the following form [18,19]:

(1) $$\begin{array}{l} \sum_{j^{\prime }{m^{\prime }}_{j}K^{\prime }}\langle 〈jm_{j}\vec{k}K|T+T_{s}+V_{A}+V_{H}+V_{\mathrm{HET}}\\ \;+V_{\mathrm{XC}}|j^{\prime }{m^{\prime }}_{j}\vec{k}K\rangle \langle j^{\prime }{m^{\prime }}_{j}\vec{k}K|v\vec{k}\rangle \\ \;=E\left(\vec{k}\right)\langle jm_{j}\vec{k}K|v\vec{k}\rangle \text{,} \end{array}$$

where *T* is the unperturbed kinetic energy term generalized for a heterostructure, *T*_*S*_ is the strain energy term that originated from the lattice mismatch*, V*_*HET*_ is the square potential due to the difference between energy gaps, *V*_*XC*_ is the exchange-correlation potential, *V*_*H*_ is the Hartree potential, and *V*_*A*_ is the ionized acceptor potential [18-20]. The Luttinger parameters as well as the other terms appearing in the secular equation are to be taken for each epitaxial layer of the SL and were extracted from [18-21]:

(2) $$\begin{array}{l} \langle 〈jm_{j}K|V_{H}+V_{A}|j^{\prime }{m^{\prime }}_{j}K^{\prime }〉\rangle =\frac{-4\pi e^{2}}{\varepsilon |K-K^{\prime }|{}^{2}}\langle 〈K|p\left(z\right)\\ \;-N_{A}|K^{\prime }〉\rangle \delta_{jj^{\prime }}\delta_{m_{j}{m^{\prime }}_{j}}\text{,} \end{array}$$

with *N*_*A*_ being the acceptor doping concentration and *p*(*z*) the hole charge distribution which is given by the following:

(3) $$p\left(z\right)=\sum_{jm_{j}k\in \text{empty}}|\langle 〈zs|jm_{j}\vec{k}〉\rangle |{}^{2}\text{.}$$

The exchange-correlation potential contribution within LDA is taken into account as in our previous works; therefore, details can be found elsewhere [22,23].

From the calculated eigenstates, one can determine the luminescence spectra of the systems by applying the following general expression [24]:

(4) $$\begin{array}{l} I\left(\omega \right)=\frac{2\hbar \omega^{3}}{c}\frac{e^{2}}{m_{0}c^{2}}\sum_{k}\sum_{n_{e}}\sum_{\begin{array}{c} n_{q}\\ q=hh,lh,so \end{array}}f_{n_{e}n_{q}}\left(k\right)N_{n_{e}k}\left[1-N_{n_{q}k}\right]\\ \;\times \frac{1}{\pi }\frac{\gamma_{n_{e}k\;n_{q}k}}{{\left[E_{n_{e}}\left(k\right)-E_{n_{q}}\left(k\right)-\hbar \omega \right]}^{2}+\gamma_{n_{e}k\;n_{q}k}^{2}}\text{,} \end{array}$$

where *e* is the electron charge, *m*_0_ is its mass, *ω* is the incident radiation frequency, *γ* is the emission broadening, *n*_*e*_ and *n*_*q*_ are the electron and hole states associated to the transition, and $E_{n_{e}}$ and $E_{n_{q}}$ are the energies associated to them. $N_{n_{e}k}$ and $\left[1-N_{n_{q}k}\right]$ are the Fermi-like occupation functions for the states in conduction and valence bands, respectively. The oscillator strength, $f_{n_{e}n_{q}}\left(k\right)$, is given by the following:

(5) $$f_{n_{e}n_{q}}\left(k\right)=\frac{2}{m_{0}}\sum_{\sigma_{e}\sigma_{q}}\frac{{\left|\langle n_{e}\sigma_{e}k|p_{x}|n_{q}\sigma_{q}k\rangle \right|}^{2}}{E_{n_{e}}\left(k\right)-E_{n_{q}}\left(k\right)}\text{,}$$

where *p*_*x*_ is the dipole momentum in the direction *x*; σ_*e*_ and σ_*q*_ denote the spin values for electrons and holes, respectively. We consider the gap energy for InGaAsN alloys as described in [12]. We also used an approach for different temperatures, considering the Varshni correction as given in [25]. However, it is important to note that for the reported high concentrations of In (0.25 to 0.41) and N (0 to 0.052) at low temperatures (*T* < 60 K), the PL spectra shows an energy blueshift, mainly due to the recombination of excitons localized most likely in the In-N clusters [26].

## Results and discussion

**Figure 1:**
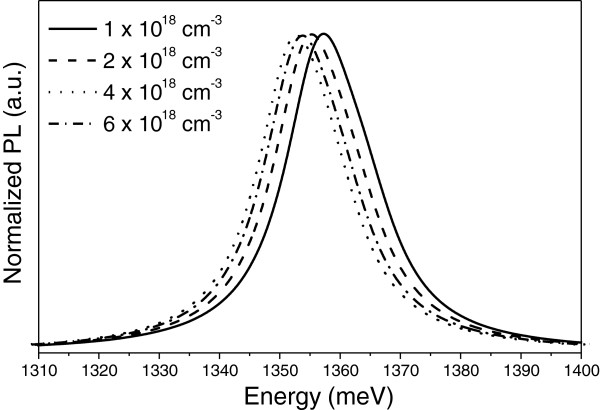
**Theoretical PL spectra, at 2 K, for unstrained p-doped GaAs/In**_**x**_**Ga**_**1−*x***_**As**_**1−*y***_**N**_***y***_**SL.** With *x* = 3%, *y* = 1.3%, barrier width, *d*_1_ = 3 nm, and well width, *d*_2_ = 3 nm. The acceptor concentration is varied for *N*_*A*_ = 1 × 10^18^ cm^−3^ (solid line), 2 × 10^18^ cm^−3^ (dashed line), 4 × 10^18^ cm^−3^ (dotted line), and 6 × 10^18^ cm^−3^ (dot-dashed line).

**Figure 2:**
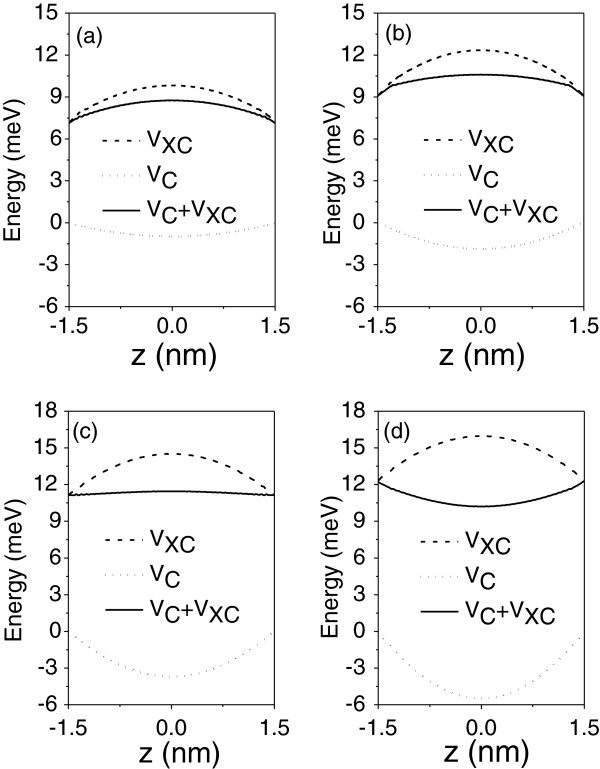
**Different contributions to the self-consistent heavy-hole potential for the same system of Figure**1**.** With **(a)***N*_*A*_ = 1 × 10^18^ cm^−3^, **(b)***N*_*A*_ = 2 × 10^18^ cm^−3^, **(c)***N*_*A*_ = 4 × 10^18^ cm^−3^, and **(d)***N*_*A*_ = 6 × 10^18^ cm^−3^. Dotted line indicates the Coulomb potential, *V*_*C*_; dashed line indicates the exchange-correlation potential, *V*_*XC*_, and solid line indicates the total potential give by the sum of *V*_*C*_ and *V*_*XC*_.

Figure 1 shows the PL spectra at *T* = 2 K for p-type GaAs/In_*x*_Ga_1−*x*_As_1−*y*_N_*y*_ SL with *x* = 3%, *y* = 1.3%, barrier width, *d*_1_ = 3 nm, and well width, *d*_2_ = 3 nm. From the literature [10,11,13], one can estimate the order of magnitude of hole concentrations, *N*_*A*_. Four different hole concentrations, *N*_*A*_, of this same order of were used, and they are 1 × 10^18^, 2 × 10^18^, 4 × 10^18^, and 6 × 10^18^ cm^−3^. The systems present strain in the barrier as well as in the well though they are compensating each other. The peak in the spectra is assigned to the first electronic transition, from electron (E1)- to the heavy hole (HH1)-confined state. The notation indicates the first level occupied for each carrier. We observe a redshift in energy as the concentration increases, and after the value of *N*_*A*_ = 4 × 10^18^ cm^−3^, we see a blueshift. This behavior is due to the different contributions for the Coulomb (*V*_*C*_) and exchange-correlation potentials (*V*_*XC*_) to the total potential, explained as follows. The competition between these potentials can generate a repulsive or attractive bending in the total potential since their sum will determine the shape of this bending inside the well. Thus, the energy levels lie near or far from the top of the valence band, decreasing or increasing the electronic transition. For a better comprehension, we present in Figure 2 the self-consistent heavy-hole (ground state) potential profiles inside the well for the same systems described above. Clearly, it is possible to see that for *N*_*A*_ = 1 × 10^18^ cm^−3^ and for *N*_*A*_ = 2 × 10^18^ cm^−3^, *V*_*XC*_ plays a major role in comparison with *V*_*C*_, so the total potential has an attractive profile. This is a consequence of the charge-density localization, which is mostly concentrated at the well center. Therefore, since the exchange-correlation potential depends on the local charge density, it is expected that this one dominates over the Coulomb potential. For *N*_*A*_ = 4 × 10^18^ cm^−3^, both potentials are practically the same, and the bending is almost flat. Above this concentration, the bending acquires a repulsive behavior. In this case, the Coulomb potential is more significant than the exchange-correlation potential.

**Figure 3:**
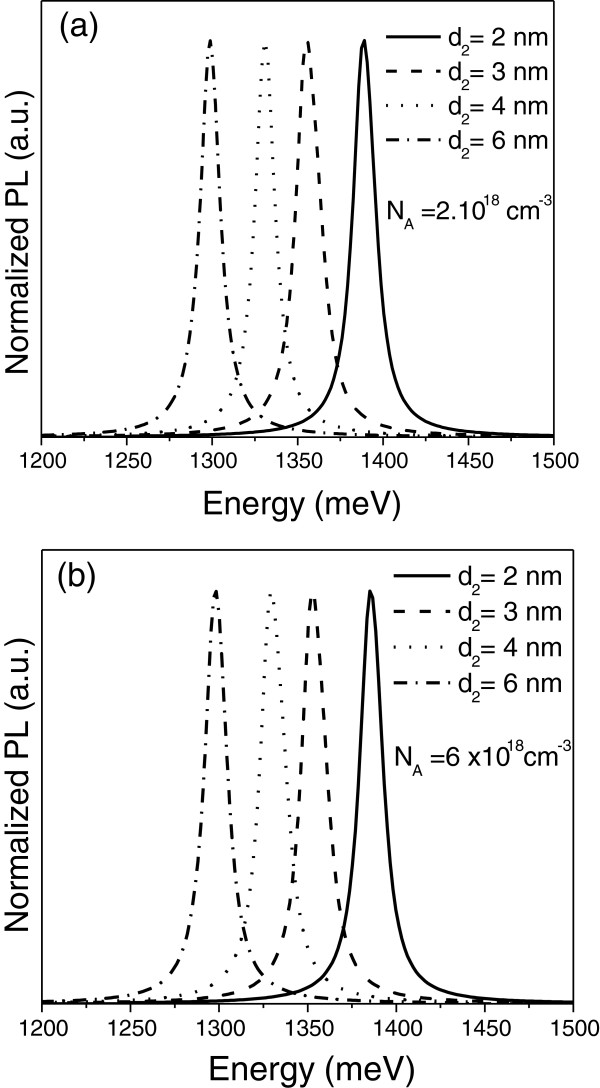
**Theoretical PL spectra at*T*=2 K for the same system described in Figure**1**.** With fixed *d*_1_ = 3 nm for **(a)***N*_*A*_ = 2 × 10^18^ cm^−3^ and (**b**) *N*_*A*_ = 6 × 10^18^ cm^−3^. The well width is varied for *d*_2_ = 2 nm (solid line), 3 nm (dashed line), 4 nm (dotted line), and 6 nm (dot-dashed line).

In Figure 3, we analyze the PL spectra at *T* = 2 K by changing the well width, *d*_2_ = 2, 3, 4, and 6 nm, for a fixed barrier *d*_1_ = 3 nm for the same structures described above with *N*_*A*_ = 2 × 10^18^ cm^−3^ and *N*_*A*_ = 6 × 10^18^ cm^−3^. In both cases, we observe a redshift in energy as the well width increases. The character of the bending, repulsive or attractive, in the total potential profile remains unchanged in both cases; the levels are just closer to the top of the valence band as the well width increases, decreasing the optical transition.

**Figure 4:**
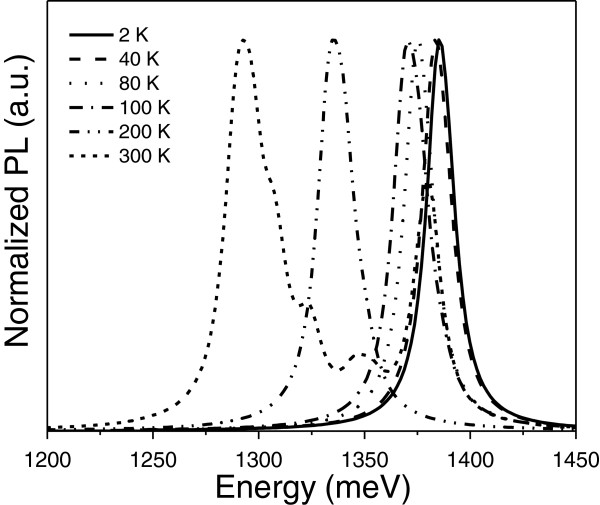
**Temperature dependence of the normalized calculated PL spectra as obtained in Figure**1**.** With *d*_1_ = 3 nm, *d*_2_ = 2 nm, and *N*_*A*_ = 6 × 10^18^ cm^−3^. From *T* = 2 K to *T* = 200 K, we have two peaks with close energies, which correspond to E1-HH1 and E1-LH1 electronic transitions. After that, for *T* = 300 K, there appear three more peaks, in addition to the first two lowest peaks, which are ascribed to the recombination involving the other excited hole states.

The effects of temperature are analyzed in Figure 4, in which we show the calculated PL spectra as a function of temperature for the same system of Figure 3 with *d*_1_ = 3 nm and *d*_2_ = 2 nm and for *N*_*A*_ = 6 × 10^18^ cm^−3^. There is a redshift in the position of the lowest peak of the spectra as the temperature increases. The first peak, as cited previously, corresponds to the first electronic transition, from electron (E1) to the heavy hole (HH1). The second peak is associated with the second transition, E1-LH1, with LH1 being the first light hole level. Actually, the first and second peaks are almost indistinguishable because the energy levels are very close. This fact occurs from *T* = 2 K up to *T* = 200 K. After that and for *T* = 300 K, we have the two lowest peaks, E1-HH1 and E1-LH1. Here, they are separated by a more significant amount of energy, followed by three more peaks, which correspond to E1-HH2, E1-HH3, and E1-SO1 (first split-off hole level), respectively. The latter shows a stronger peak due to a larger oscillator strength, which is larger than the superposition of the wave functions of the second, third, and fourth states in the valence and conduction bands. As the temperature increases to 300 K, the main peak spans from transitions to the fundamental state to transitions to the first excited state and so on, giving rise to the multiple peaks seen. The redshift observed in the spectra is related to the InGaAsN gap shrinkage, according to the Varshni approximation [25].

## Conclusions

We present here for the first time the theoretical PL spectra for GaAs/InGaAsN systems obtained using self-consistent effective mass theory calculations. We noted a remarkable change in the total potential when the acceptor concentration increases. For the cases discussed here, changes in the well width do not change the shape of bending for the total potential. Furthermore, and as expected, we see a redshift in the PL spectra as the temperature increases. The present results show that in modulation p-doped GaAs/InGaAsN nanostructures, the many-body effects, such as exchange and correlation, must be taken into account for a realistic description of hole bands and potentials in these systems. These findings will certain have important implications for optical measurements, such as luminescence or absorption, towards developing new technologies based on nanostructured superlattices. This will be important in the development of new optoelectronic devices, solar cells, and other devices.

## Competing interests

The authors declare that they have no competing interests.

## Authors’ contributions

TFO carried out the calculations. GMS, LMRS and EFSJ discussed the results and proposed new calculations and improvements. SCPR conceived the study and participated in its design and coordination. All authors read and approved the final manuscript.
